# Comprehensive Genome-Wide Investigation and Transcriptional Regulation of the bZIP Gene Family in Litchi Fruit Development

**DOI:** 10.3390/plants14101453

**Published:** 2025-05-13

**Authors:** Jiaxuan Liu, Saimire Silaiyiman, Jiaxin Wu, Lejun Ouyang, Zheng Cao, Chao Shen

**Affiliations:** 1 Guangdong Provincial Key Laboratory for Green Agricultural Production and Intelligent Equipment, College of Biological and Food Engineering, Guangdong University of Petrochemical Technology, Maoming 525000, China; 18375432965@163.com (J.L.); 19990885586@163.com (S.S.); 15077668902@163.com (J.W.); ouyanglejun@gdupt.edu.cn (L.O.); 2 College of Life and Geographic Sciences, Kashi University, Kashi 844000, China; 3 Key Laboratory of Biological Resources and Ecology of Pamirs Plateau in Xinjiang Uygur Autonomous Region, Kashi 844000, China; 4 Maoming Agricultural Science and Technology Extension Center, Maoming 525000, China; caozheng5192@163.com

**Keywords:** litchi chinensis, bZIP family, transcriptional regulation, fruit development

## Abstract

*Litchi chinensis*, a crucial tropical and subtropical fruit tree in southern China, is widely appreciated for its distinctive flavor, high nutritional value, and significant economic impact. The bZIP (basic leucine zipper) gene family plays an essential role in regulating key biological functions during plant growth and development. In this study, we performed a comprehensive bioinformatics analysis of the bZIP gene family in litchi to systematically elucidate its molecular characteristics and functional properties. A total of 55 bZIP gene family members were identified, with the encoded proteins containing between 129 and 845 amino acid residues and theoretical isoelectric points (pI) ranging from 4.85 to 10.23. Protein–protein interaction network analysis revealed that 46 proteins exhibited interaction relationships. Phylogenetic analysis classified these genes into 13 distinct subgroups (A–K, M, and S). Chromosomal localization analysis indicated that bZIP gene family members were successfully mapped to 15 chromosomes. Intraspecific collinearity analysis identified 39 segmental duplication events, while interspecific and single-gene collinearity analyses suggested evolutionary conservation, with only a few genes exhibiting duplication or loss events. Cis-acting element analysis revealed a total of 213 elements associated with growth and development, which may play an important role in fruit development regulation. The results of differential gene expression, related to fruit development across different *litchi* cultivars, tissues, and flowering stages, combined with qRT-PCR validation, suggest that *LITCHI017015.m1* and *LITCHI004463.m1* may be involved in the early regulation of fruit development, while *LITCHI018843.m1* may play a regulatory role during the later stages of fruit development. These findings provide a strong theoretical foundation for understanding the roles of bZIP genes in litchi fruit growth and development, and lay the groundwork for further functional studies. This study has potential application value in litchi fruit development and genetic improvement.

## 1. Introduction

Transcription factors (TFs) are key regulatory proteins that control gene expression, typically by binding to cis-acting elements in the promoter regions of target genes to either activate or repress their transcription. Basic leucine zipper (bZIP) transcription factors are widely distributed in eukaryotes and represent one of the largest TF families in plants [1]. Structurally, bZIP proteins consist of two functional domains: a basic region at the N-terminus composed of 16–20 basic amino acids responsible for recognizing and binding to the ACGT core sequence [2,3], and a leucine zipper region at the C-terminus composed of heptad repeat units that facilitate dimer formation. These dimers exhibit high specificity, with members of the same subfamily tending to form homodimers, while those from different subfamilies may form heterodimers. The formation and function of these dimers can vary under different environmental conditions and are also influenced by post-translational modifications such as phosphorylation [4]. Additionally, some plant bZIP proteins contain transcriptional activation domains enriched in proline, glutamine, or acidic amino acids [5]. The “Y”-shaped structure of the bZIP dimers enables precise binding to cis-elements in the promoters of target genes, thereby regulating gene expression and participating in various biological processes [6]. bZIP transcription factors have been identified in numerous plant species, including *Arabidopsis thaliana* [7], rice (*Oryza sativa*) [8], sorghum (*Sorghum bicolor*) [9], melon (*Cucumis melo*) [10], poplar (*Populus tremula*) [11], and rapeseed (*Brassica napus*) [12], providing a theoretical foundation for understanding their functions and the evolutionary processes of plants.

Studies have found that bZIP transcription factors play an important role in plant growth and development, regulating responses to abiotic stresses such as drought, high salinity, and low temperature in plants like *Arabidopsis thaliana* [13], rice (*Oryza sativa*) [14], wheat (*Triticum aestivum*) [15], tomato (*Solanum lycopersicum*) [16], pepper (*Capsicum annuum*) [17], soybean (*Glycine max*) [18], and maize (*Zea mays*) [19]. They are also involved in organ development and formation, covering critical stages such as seed maturation and germination, embryo development, flowering, and photomorphogenesis [20]. For example, in wheat, candidate bZIP genes regulate starch biosynthesis [21], while in rice, the OsbZIP76 mutant exhibits a reduced seed size and lower amylose content [22]. Additionally, bZIP transcription factors are involved in plant adaptation to extreme environmental conditions [23], such as AabZIP1, which activates ADS and CYP71AV1 to promote artemisinin synthesis in *Artemisia annua* [24].

In various signal transduction pathways, bZIP proteins regulate responses to ABA, light, osmotic stress, and pathogen infection, which are crucial for plant adaptation to adversity [25,26]. bZIP proteins also play a role in organ differentiation, cell elongation, carbon and nitrogen metabolism, unfolded protein response, and seed gene expression regulation [27]. For instance, AaAPK1 can phosphorylate AabZIP1 to enhance its transcriptional activation function [28], and the overexpression of AabZIP9 promotes artemisinin accumulation [29]. Furthermore, LV et al. found that AaTGA6 mediates salicylic acid signaling through AaERF1, regulating artemisinin synthesis [30], further highlighting the multifunctionality of bZIP factors.

In fruit ripening, bZIP transcription factors also play a central role. In grape (*Vitis vinifera*),, the *VvABF2* is significantly correlated with grape berry ripening, acting as a regulatory factor of ABA-dependent [31]. In peaches, certain bZIP genes may function as “rhythmizers” in the metabolic pathways regulating ripening [32]. Moreover, bZIP genes can influence amino acid and sugar metabolism, improving fruit flavor and quality [33]. For example, S1-bZIPs (such as tbz17 and AtbZIP11 mORF) have been shown to increase sucrose concentration in transgenic plants [34]. In tomatoes, SlbZIP1 and SlbZIP2 also enhance fruit sweetness without negative effects on the plant [35].

*Litchi chinensis*, a tropical evergreen tree in the Sapindaceae family, is widely distributed in subtropical regions of southern China and is popular for its unique flavor and rich nutritional value [36,37]. Its cultivation history dates back over two thousand years to the Han Dynasty. In addition to its high culinary value, litchi also exhibits antioxidant, anti-inflammatory, and health-promoting properties due to its polyphenolic and flavonoid compounds [38], and it has widespread use in traditional Chinese medicine.

Although bZIP transcription factors have been extensively studied in model plants, research on their roles in *Litchi chinensis* remains relatively scarce. Litchi growth depends on warm and humid climates, and its fruit development is highly sensitive to abiotic stresses such as low temperature, high temperature, and drought, which pose significant challenges to the litchi industry. Therefore, an in-depth study of the structure and function of the bZIP gene family in litchi, and especially its expression patterns during fruit development, holds both theoretical and practical significance. Through systematic identification and expression analysis, this research can provide solid scientific support for improving litchi quality and ensuring the sustainable development of the industry.

## 2. Results

### 2.1. Identification, Physicochemical Property Analysis, and Subcellular Localization of Litchi bZIP Transcription Factors

In *Litchi chinensis*, a total of 55 bZIP gene family members containing a leucine zipper domain were identified (Supplementary Table S1). Physicochemical property analysis revealed that the amino acid count of bZIP transcription factors varied from 129 in *LITCHI014383.m1* to 845 in *LITCHI020028.m1*. The relative molecular weight (Mr) had a large range, from 14,967.32 (*LITCHI014383.m1*) to 92,177.38 (*LITCHI020028.m1*). The isoelectric points (pIs) of litchi bZIP transcription factors were found to range from 4.85 (*LITCHI009858.m1*) to 10.23 (*LITCHI018144.m1*), with 29 classified as acidic proteins and 25 as basic proteins. Only *LITCHI020193.m1* had a neutral pI of 7, indicating its electrical neutrality. Additionally, the instability index (II) varied between 31.61 (*LITCHI025169.m1*) and 82.1 (*LITCHI006674.m1*). The aliphatic index ranged from 46.18 (*LITCHI010080.m1*) to 91.45 (*LITCHI023292.m1*), while the grand average of hydropathicity (GRAVY) values were between −1.211 (*LITCHI014217.m1*) and −0.331 (*LITCHI005494.m1*), indicating that all identified proteins exhibited hydrophilic properties (Supplementary Table S1). Furthermore, subcellular localization analysis indicated that all litchi bZIP proteins were localized in the nucleus (Supplementary Table S1).

### 2.2. Phylogenetic Analysis of the Litchi bZIP Gene Family

**Figure 1:**
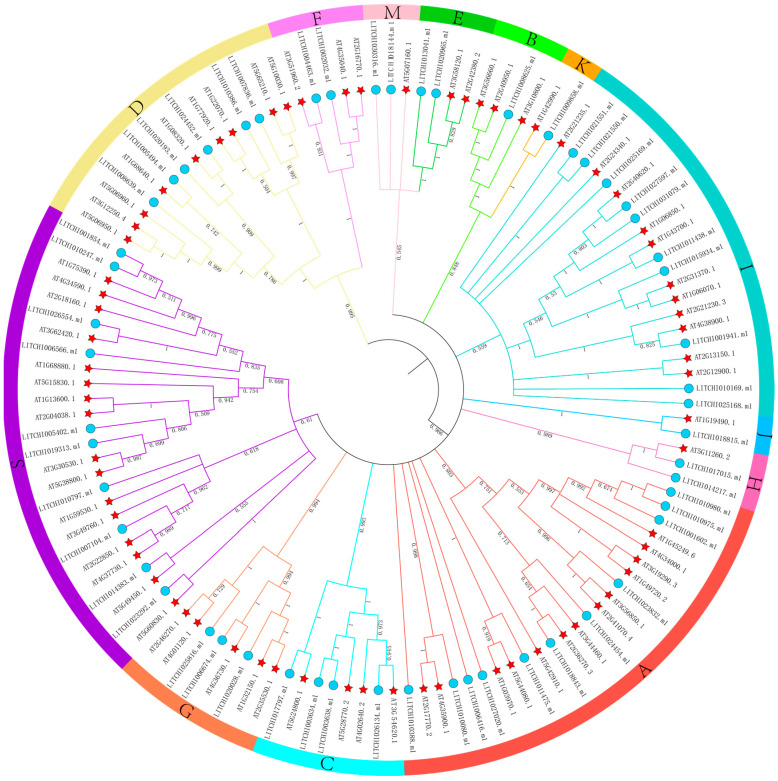
Classification and phylogenetic analysis of the bZIP gene family in *Arabidopsis thaliana* (pentagram) and *litchi* (circle). All bZIP genes were categorized into 13 classes, each class represented by a different color.

A phylogenetic tree was constructed by aligning litchi bZIP protein sequences with previously reported *Arabidopsis thaliana* bZIP proteins. Based on the classification of the *Arabidopsis* bZIP family, the litchi bZIP proteins were divided into 13 subfamilies: A, B, C, D, E, F, G, H, I, J, K, S, and M (Figure 1). Among them, subfamily A contained the highest number of litchi bZIP members (11), while subfamily B had only 1 member (*LITCHI008625.m1*). Subfamily C included 4 members and D had 6. E contained 2 (*LITCHI013041.m1* and *LITCHI020965.m1*). F had 2 (*LITCHI002032.m1* and *LITCHI004463.m1*) and G contained 3 (*LITCHI006674.m1*, *LITCHI020028.m1*, and *LITCHI025816.m1*). H had 2 (*LITCHI014217.m1* and *LITCHI017015.m1*), I included 8, J had 1 (*LITCHI018815.m1*), K had 1 (*LITCHI009858.m1*), S contained 10, and M had 2 (*LITCHI018144.m1* and *LITCHI030316.m1*). This classification lays a foundation for further functional studies on the role of bZIP transcription factors in litchi.

### 2.3. Prediction of Secondary and Tertiary Structures of Litchi bZIP Transcription Factors

To investigate the functions of the 55 bZIP transcription factor proteins in *Litchi chinensis*, we conducted secondary structure predictions. The results revealed that random coils and α-helices dominate the secondary structures of litchi bZIP transcription factors, both of which play essential roles in protein functionality (Supplementary Figure S1). Notably, bZIP members of the same subgroup showed greater structural similarity than members from different subgroups, emphasizing the evolutionary conservation of their structural features (Supplementary Figure S2).

### 2.4. Conserved Motif and Gene Structure Analysis of the Litchi bZIP Gene Family

**Figure 2:**
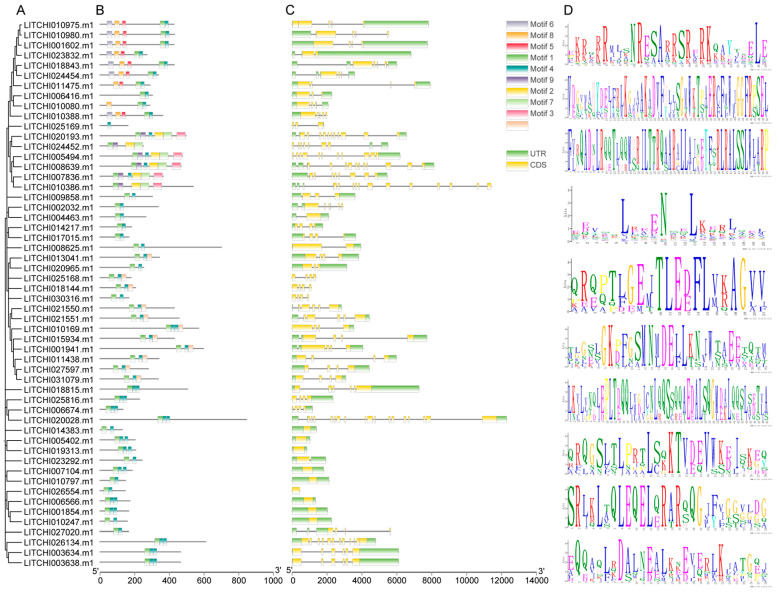
Analysis of conserved motifs and gene structure of bZIP protein in *litchi.* Phylogenetic tree of *litchi* bZIP gene family (**A**), conserved motifs (**B**), gene structure (**C**), and each amino acid sequence (**D**).

Through an in-depth analysis of the evolutionary relationships, gene structure (Figure 2), and conserved domain (Supplementary Figure S3) of the litchi bZIP gene family, we observed that members within the same subfamily exhibit a high degree of similarity in both gene structure and conserved motifs. Motif analysis revealed that most litchi bZIP genes contain motif 1, which appears to be a widely conserved sequence in this gene family and may play a crucial role in gene function and structural stability. Regarding gene structure, our analysis indicated that all litchi bZIP genes consist of both exons and introns, with exon numbers ranging from 1 (*LITCHI026554.m1*) to 18 (*LITCHI020028.m1*).

### 2.5. Chromosomal Localization and Interaction Analysis of Litchi bZIP Genes

**Figure 3:**
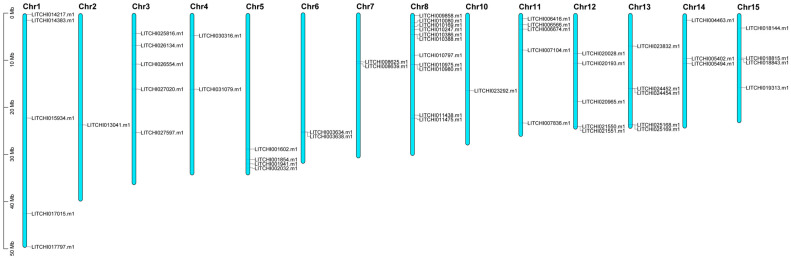
Chromosomal localization of bZIP genes in litchi.

The bZIP gene family members in *Litchi chinensis* have been successfully mapped to 15 chromosomes, with an uneven distribution pattern (Figure 3). Notably, Chr9 does not contain any bZIP genes, while Chr2 and Chr10 each harbor only one bZIP gene. By contrast, Chr4, Chr6, and Chr7 each contain two bZIP genes. Interestingly, most other chromosomes contain three or more bZIP genes. Among them, Chr8 harbors the highest number of bZIP transcription factors (11 genes), accounting for 20.0% of the total. Furthermore, the spatial distribution of bZIP genes on individual chromosomes varies. For instance, bZIP genes tend to cluster at the terminal regions of Chr5, Chr6, and Chr11, whereas their presence in the central regions is relatively sparse. By contrast, Chr3 exhibits a more evenly distributed pattern.

**Figure 4:**
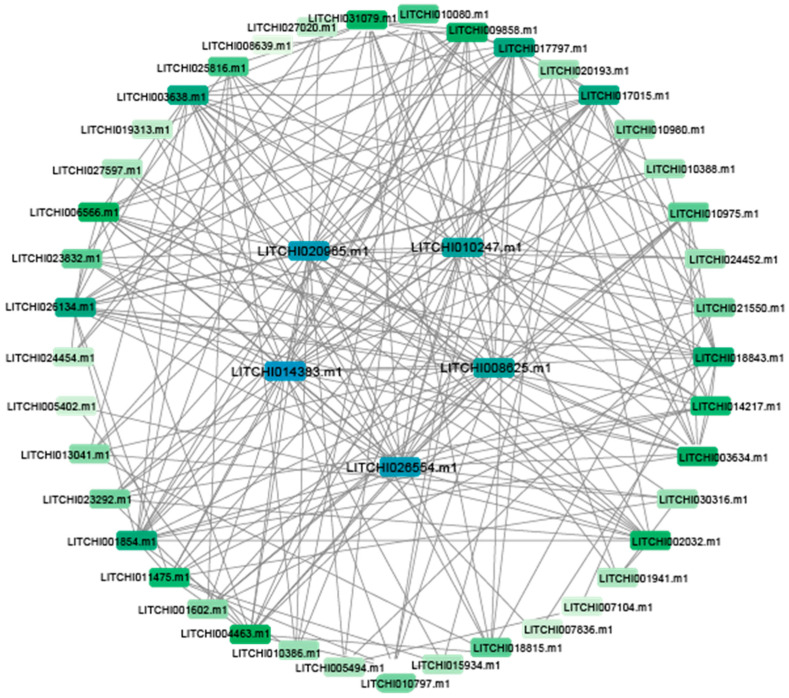
The litchi bZIP protein interaction network. Each box represents a protein. The color of the protein represents the strength of the interaction; the darker the color, the stronger the interaction. The five genes in the center are hub genes.

To investigate the functional relationships among proteins, we employed homology mapping to predict the interaction network of litchi bZIP transcription factors (Figure 4). Our study revealed interactions among 46 bZIP proteins. The number of protein interactions was mapped using a color gradient, with deeper colors indicating a higher number of interactions. Notably, *LITCHI020965.m1*, *LITCHI010247.m1*, *LITCHI014383.m1*, *LITCHI008625.m1*, and *LITCHI026554.m1* exhibited the highest interaction counts and were represented by the deepest colors. Meanwhile, other proteins also displayed interconnections within the network.

### 2.6. Intra- and Interspecies Synteny Analysis, Gene Duplication, and Divergence of Litchi bZIP Genes

**Figure 5:**
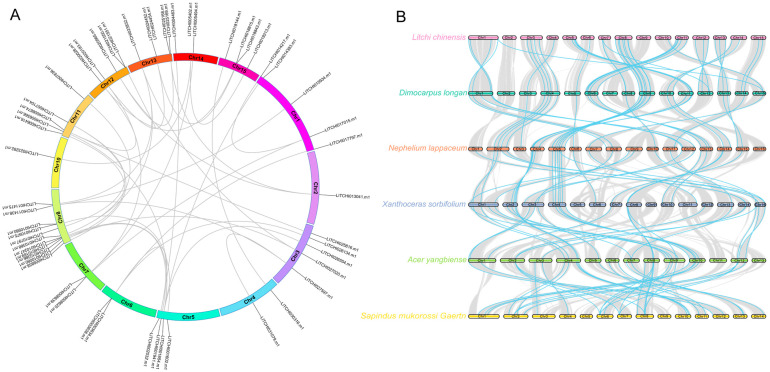
Synteny analysis of litchi bZIP genes within and across species. Collinearity analysis of *Litchi* bZIP genes (**A**) within the *Litchi* genome and (**B**) across species. The blue lines represent collinear bZIP genes.

**Figure 6:**
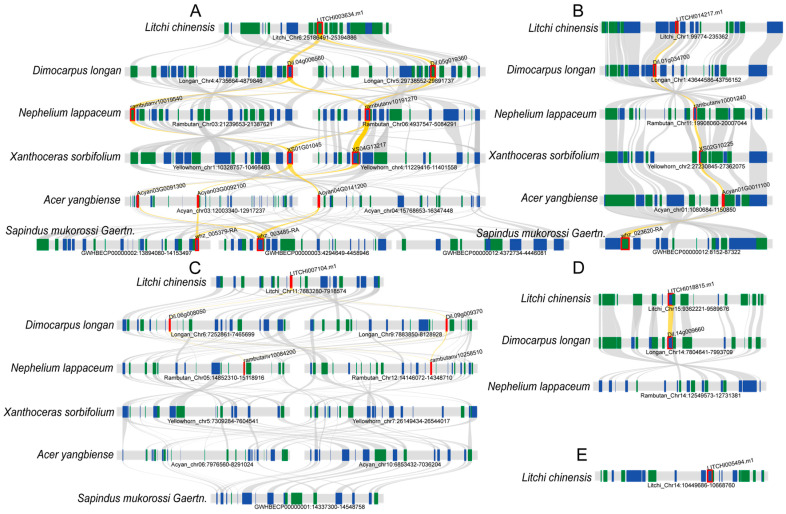
Duplication and loss events of *Sapindaceae* bZIP genes. The red boxes indicate the locations of bZIP genes, while the yellow lines represent gene collinearity. The gene is (**A**) duplicated among species; (**B**) conserved among species and is a single copy; (**C**) duplicated among some species and lost among others; (**D**) conserved among some species, is a single copy, and is lost among some species; and (**E**) unique to litchi.

The gene duplication events in the bZIP gene family revealed a high degree of homology among bZIP genes across different chromosomes. Among the 55 bZIP genes, 39 segmental duplication events were detected, with individual bZIP genes undergoing between 1 and 4 duplication events (Figure 5A). To further explore the evolutionary relationships, a synteny analysis of bZIP genes was conducted across six species within the *Sapindaceae* family, providing insights into the macro-level distribution patterns of bZIP genes in different species (Figure 5B). Focusing on the gene duplication and loss events of individual litchi bZIP genes within *Sapindaceae*, we found that *LITCHI003634.m1* and *LITCHI019313.m1* were duplicated in both litchi and its close relative *Dimocarpus longan* (Figure 6A and Supplementary Figure S4). Meanwhile, *LITCHI007104.m1* underwent duplication during the evolutionary transition from litchi to *Nephelium lappaceum*, but was subsequently lost in later evolutionary stages (Figure 6C and Supplementary Figure S5). Conversely, some bZIP genes, such as *LITCHI014217.m1* and *LITCHI026134.m1*, remained as single-copy genes, with no observed duplication or loss throughout evolution (Figure 6B and Supplementary Figure S6). By contrast, genes such as *LITCHI018815.m1* and *LITCHI005494.m1* were lost in litchi or its closely related species (Figure 6D,E and Supplementary Figure S7). These findings highlight the complex evolutionary history of bZIP genes in *Litchi chinensis* and its relatives.

### 2.7. Prediction of Cis-Acting Elements in the Litchi bZIP Gene Family

**Figure 7:**
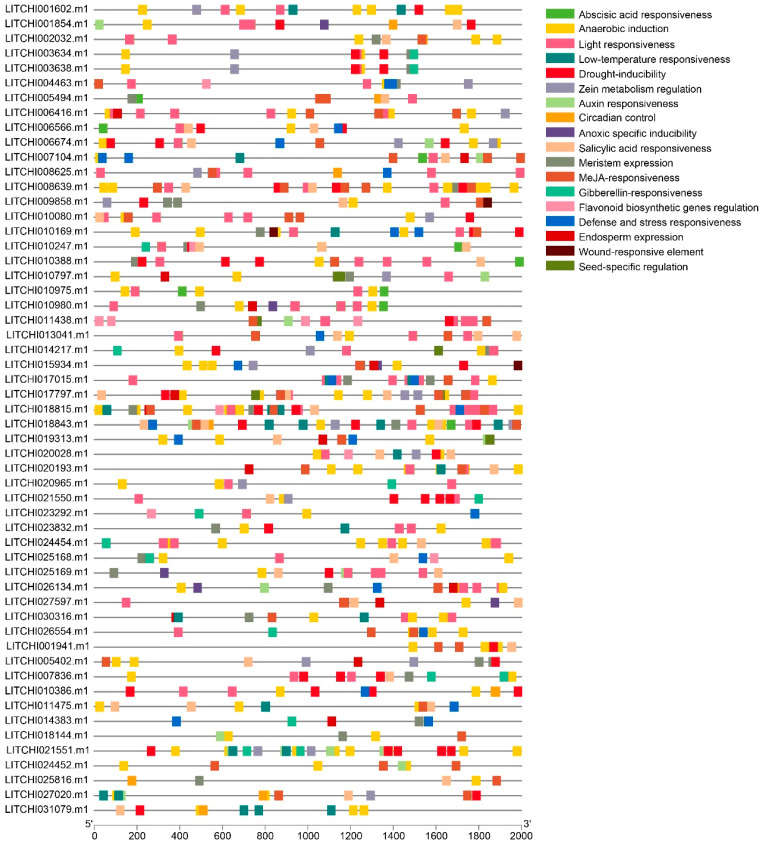
An analysis of the cis-regulatory elements of bZIP gene family promoters in litchi. The scale at the bottom indicates the length of the sequence. Different cis-acting elements are represented by different colors.

In this study, cis-acting elements were extracted from the 2000 bp upstream regions of the litchi bZIP gene coding sequences (Figure 7), and they were classified into four major categories: light-responsive elements, plant growth and developmental regulation elements, plant-hormone-responsive elements, and stress-responsive elements. Among the light-responsive elements, 369 elements were identified, including ACE and G-Box elements. The plant-hormone-responsive elements were the most diverse, with 514 elements detected, such as ARBE (abscisic acid response), AuxRR-core (auxin response), MBSI (flavonoid biosynthesis regulation), TATC-box (gibberellin response), and TCA-element (salicylic acid response). The stress-responsive elements were the most abundant, totaling 723 elements, with key elements including TC-rich repeats (defense and stress response), MBS (drought response), LTR (low-temperature response), ARE (anaerobic response), and WUN-motif (mechanical injury response). In terms of regulatory elements of plant growth and development, 213 elements were identified, including circadian (circadian rhythm regulation), GCN4_motif (endosperm expression), CAT-box (meristem expression), RY-element (seed-specific regulation), and O2-site (zein metabolism regulation). These results suggest that the *bZIP* gene family in litchi plays an essential role in environmental adaptation, hormone signaling, and plant developmental processes.

### 2.8. Tissue Expression Patterns of Litchi bZIP Genes and Their Differential Expression in Various Fruit Developmental Stages

The tissue-specific expression patterns of bZIP genes in litchi are crucial for understanding their potential functions within the genome. In this study, we analyzed 616 GB of transcriptomic data from various litchi tissues, revealing widespread expression of the bZIP genes across different tissues.

**Figure 8:**
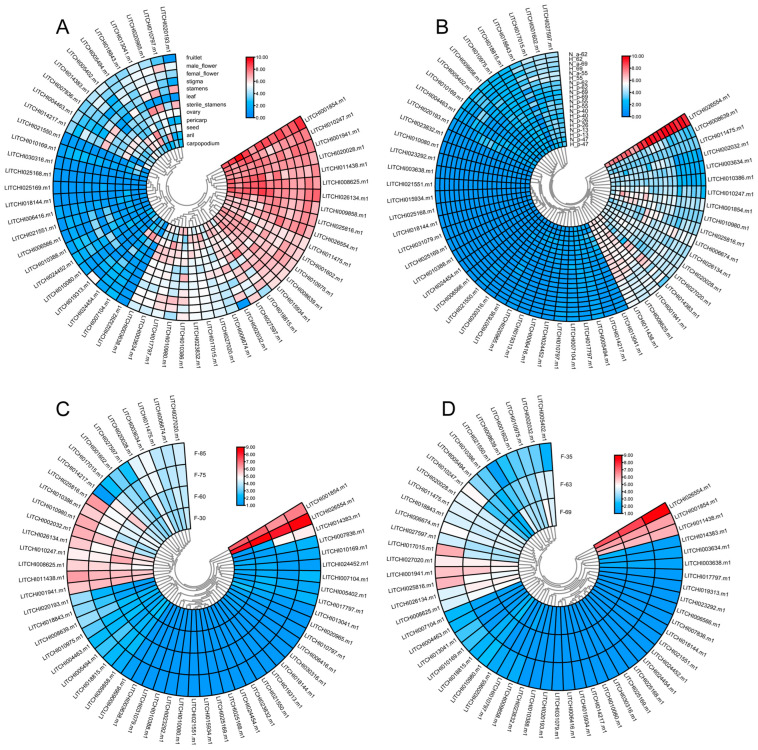
Tissue expression patterns of the litchi bZIP gene family across different tissues, species, and developmental stages after flowering. Expression analysis of bZIP genes (**A**) in different tissues of litchi, (**B**) in the pericarp and aril of Nuomici and Huaizha in different post-anthesis stages, (**C**) in litchi pericarp in different stages after flowering, and (**D**) in litchi “Feizixiao” in different stages after flowering.

Among them, *LITCHI001854.m1*, *LITCHI026554.m1*, *LITCHI010247.m1*, and *LITCHI001941.m1* exhibited higher expression levels compared to other genes (Figure 8A). Furthermore, during the fruit flesh and peel developmental stages at 55, 62, and 69 days after anthesis in the two cultivars *Nuomici* and *Huaizha*, the genes *LITCHI006654.m1, LITCHI028625.m1*, and *LITCHI001854.m1* showed significantly higher expression, while most other bZIP genes displayed relatively low expression levels (Figure 8B). During fruit peel development, *LITCHI006654.m1*, *LITCHI001854.m1*, and *LITCHI011438.m1* exhibited the highest expression among the bZIP genes (Figure 8C). In the study of the “sugar receding” phenomenon in *Feizixiao* litchi pulp, we analyzed the expression patterns of bZIP genes at 35, 63, and 69 days after anthesis. The results showed that *LITCHI006654.m1, LITCHI001854.m1*, and *LITCHI011438.m1* consistently exhibited high expression levels across all three time points (Figure 8D). A comprehensive analysis of bZIP gene expression patterns across different cultivars, developmental stages, and tissues revealed that *LITCHI006654.m1* maintained high expression throughout fruit development, followed by *LITCHI001854.m1*, suggesting that these two genes play crucial roles in litchi tissue differentiation and fruit development.

**Figure 9:**
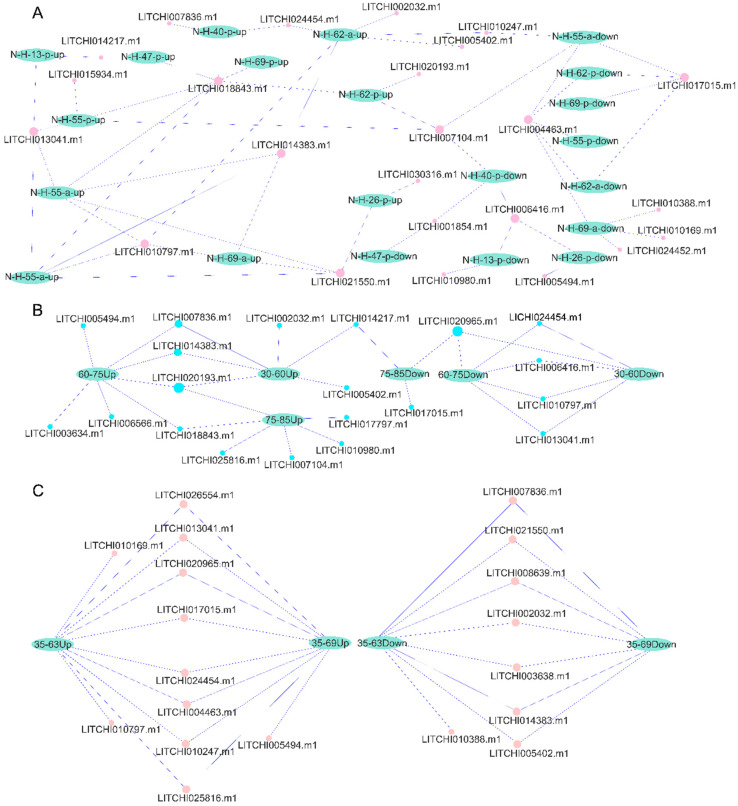
Up- and downregulated expressions of bZIP genes in different litchi cultivars, tissues, and flowering stages. The differential expression of bZIP genes (**A**) in the pericarp and aril of *Nuomici* and *Huaizha* in different post-anthesis stages, (**B**) during litchi pericarp development at 30–60, 60–75, and 75–85 days after flowering, and (**C**) in the *Feizixiao* cultivar 35–63, 35–69, and 63–69 days after flowering. “N” represents the species *Nuomici*, “H” represents the species *Huaizha*, “a” represents aril, and “p” represents pericarp. The numbers indicate days after flowering. “Up” indicates upregulated expression and “Down” indicates downregulated expression.

To further investigate the differential expression of bZIP genes during litchi fruit development, we analyzed the expression patterns of bZIP genes in the pericarp and aril of two litchi cultivars, *Nuomici* and *Huaizha*, in different post-anthesis stages (Figure 9A, Supplementary Figure S8A, Supplementary Table S2). We found that the genes *LITCHI017015.m1* and *LITCHI004463.m1* exhibited downregulated expression in the pericarp and aril of *Nuomici* and *Huaizha* at 55, 62, and 69 days after flowering (DAF), while *LITCHI006416.m1* also exhibited downregulated expression in the pericarp at 13, 26, and 40 DAF. Conversely, the genes *LITCHI014383.m1*, *LITCHI018843.m1*, *LITCHI013041.m1*, *LITCHI010797.m1*, and *LITCHI021550.m1* exhibited upregulated expression across different developmental stages in both the pericarp and aril of *Nuomici* and *Huaizha* (Figure 9A, Supplementary Figure S8A, Supplementary Table S2).

During litchi pericarp development, we further analyzed the differential expression of bZIP genes in three distinct time periods: 30–60, 60–75, and 75–85 DAF (Figure 9B, Supplementary Figure S8B, Supplementary Table S3). In the 30–60 DAF period, five genes, including *LITCHI020193.m1* and *LITCHI014383.m1*, exhibited upregulated expression, while five genes, including *LITCHI006416.m1* and *LITCHI010797.m1*, exhibited downregulated expression. During the 60–75 DAF period, six genes, including *LITCHI020193.m1* and *LITCHI018843.m1*, exhibited upregulated expression, whereas five genes, including *LITCHI013041.m1* and *LITCHI024454.m1*, exhibited downregulated expression. In the 75–85 DAF period, six genes, including *LITCHI020193.m1* and *LITCHI017797.m1*, exhibited upregulated expression, while three genes, including *LITCHI020965.m1* and *LITCHI014217.m1*, exhibited downregulated expression. Notably, *LITCHI020193.m1* exhibited upregulated expression across all three periods, while *LITCHI020965.m1* exhibited downregulated expression consistently in all three periods.

In the study of the “sugar receding” phenomenon in the pulp of the *Feizixiao* cultivar, we analyzed gene expression changes during the 35–63, 35–69, and 63–69 DAF periods (Figure 9C, Supplementary Figure S8C, Supplementary Table S4). During the 35–63 DAF period, ten genes, including *LITCHI010169.m1* and *LITCHI010797.m1*, exhibited upregulated expression, while eight genes, including *LITCHI010388.m1* and *LITCHI007836.m1*, exhibited downregulated expression. In the 35–69 DAF period, nine genes, including *LITCHI005494.m1* and *LITCHI025816.m1*, exhibited upregulated expression, whereas seven genes, including *LITCHI005402.m1* and *LITCHI007836.m1*, exhibited downregulated expression. However, no differentially expressed bZIP genes were detected in the 63–69 DAF period.

### 2.9. qRT-PCR Expression Analysis of the bZIP Genes

**Figure 10:**
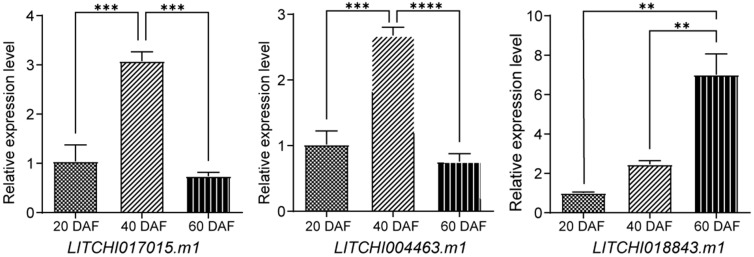
Fluorescence quantitative expressions of the bZIP gene. Asterisks indicate significant differences based on one-way ANOVA (** *p* < 0.01; *** *p* < 0.001; **** *p* < 0.0001).

Based on the differential expression analysis of bZIP genes across various litchi cultivars, tissues, and flowering stages during fruit development, we hypothesize that *LITCHI017015.m1* and *LITCHI004463.m1* may be involved in the regulation of early fruit development, whereas *LITCHI018843.m1* may play a role during the later stages of fruit maturation. To validate this hypothesis, we conducted quantitative real-time PCR (qRT-PCR) analysis on the three bZIP candidate genes (*LITCHI017015.m1*, *LITCHI004463.m1*, and *LITCHI018843.m1*) to examine their expression patterns throughout the fruit developmental process. The qRT-PCR results revealed that *LITCHI017015.m1* and *LITCHI004463.m1* were significantly upregulated between 20 and 40 days after flowering (DAF), corresponding to the early stage of fruit development, while their expression levels declined notably by 60 DAF. These results suggest that both genes may play important regulatory roles in the early phase of fruit development (Figure 10). By contrast, *LITCHI018843.m1* exhibited low expression levels at 20 and 40 DAF, but was significantly upregulated at 60 DAF, indicating its potential involvement in the regulation of late-stage fruit development (Figure 10).

## 3. Discussion

The bZIP transcription factor family is one of the most conserved and widely distributed transcription factor families in the plant kingdom. These factors play a crucial role in regulating key physiological processes such as plant growth and development, stress responses, seed germination, and senescence [39]. The bZIP gene family varies in size across different plant species [40], with the smallest family sizes found in lower plants [41]. For example, *Chlamydomonas reinhardtii* contains 17 bZIP genes, whereas *Physcomitrella patens*, one of the earliest known land mosses, has 43 bZIP genes [42]. In angiosperms, most plant genomes contain more than 40 bZIP genes [41]. In this study, based on whole-genome data for litchi, we identified a total of 55 bZIP transcription factors using bioinformatics approaches. In recent years, bZIP family members have been identified and reported in multiple species, including *Arabidopsis thaliana* (78 genes) [43], *Vitis vinifera* (55 genes) [44], *Phaseolus vulgaris* (84 genes) [45], *Malus domestica* (114 genes) [46], *Citrullus lanatus* (62 genes) [47], and *Zea mays* (54 genes) [48]. We found that the number of bZIP genes in litchi differs from that in other plant species, reflecting variations in gene family size among different plants. Subsequently, we conducted a comprehensive bioinformatics analysis of these gene family members to elucidate their characteristics.

Through the analysis of physicochemical properties, we found that all bZIP proteins in litchi are hydrophilic. Additionally, subcellular localization analysis revealed that all bZIP proteins in litchi are localized in the nucleus, consistent with findings in maize [48]. Furthermore, the secondary structure analysis of the 55 bZIP family members in litchi showed that their structures are primarily composed of random coils and α-helices. Phylogenetic analysis further classified these 55 bZIP genes into 13 subgroups, with the A and S subfamilies containing the highest number of bZIP genes. By comparison, the number of subgroups in other species varies, such as 10 in apple [46] and 11 in grape [44]. The increased number of subgroups in litchi suggests an expansion of the bZIP gene family in this species. The interspecies phylogenetic tree of litchi and Arabidopsis thaliana indicates that the bZIP gene family members in litchi share evolutionary similarities with those in Arabidopsis, suggesting their conserved nature.

Differences in gene structure organization may indicate distinct evolutionary trajectories of bZIP genes [49]. The number of exons and introns plays a crucial role in plant adaptation and developmental processes [50]. Further motif analysis revealed that most bZIP genes contain motif 1, which has also been observed in *Brassica napus* [12], suggesting its high conservation in protein structure and its crucial role in maintaining gene function and structural stability. Among the 55 identified bZIP genes, 39 segmental duplication events were detected, with duplication frequencies ranging from one to four occurrences per gene. By contrast, a study in maize [48] identified only 18 segmental duplications, significantly fewer than in litchi. However, no tandem duplication events were detected, indicating that segmental duplication is the primary driver of bZIP gene family expansion in litchi. This finding aligns with the analysis of bZIP genes in *Cannabis sativa* [51]. Similarly, in *Nephelium lappaceum* (rambutan), another species in the Sapindaceae family, segmental duplication has been demonstrated to play a crucial role in the expansion of resistance genes [52].

By analyzing the duplication events of individual bZIP genes in litchi, we found that only a few *bZIP* genes underwent duplication, while the majority remained as single-copy genes, such as *LITCHI014217.m1* and *LITCHI026134.m1*. This observation highlights the functional conservation of the *bZIP* gene family across species, which is consistent with previous litchi genome studies. These studies suggest that litchi, longan (*Dimocarpus longan*), and Xanthoceras sorbifolium share a γ-triplication event but have not undergone independent whole-genome duplication (WGD) events since then [53].

Gene expression patterns are one of the key indicators for elucidating gene function [54]. Tissue-specific expression analysis revealed significant differences in the expression levels of 55 bZIP genes across different tissues. For example, *LITCHI013041.m1* exhibited high expression in seeds and arils, whereas *LITCHI007104.m1* was predominantly expressed in seeds but showed low or nearly undetectable expression in other tissues. Notably, *LITCHI001854.m1* displayed consistently high expression levels across all tissues, suggesting its broad regulatory role in litchi growth and development. Further comprehensive analysis of bZIP gene expression across different litchi cultivars and developmental stages after anthesis revealed that *LITCHI006654.m1* maintained consistently high expression throughout fruit development. Additionally, *LITCHI006654.m1* exhibited the highest number of protein interactions, indicating its potential role as a key regulatory factor in litchi fruit development. This suggests that *LITCHI006654.m1* functions both as a highly expressed gene involved in fruit development and as a central hub in the protein interaction network, regulating multiple related biological processes.

The bZIP gene family has been demonstrated to play a crucial role in fruit development across various plant species. For instance, the bZIP gene *OebZIP22* has been shown to regulate fruit development in olives [55], while *PpbZIP44h* in pears controls the expression of genes related to fruit quality compounds [56]. However, research on the role of bZIP genes in litchi fruit development remains limited.

To further investigate the impact of bZIP genes on litchi fruit development, we integrated transcriptome data from different litchi tissues and analyzed the differential expression of *bZIP* genes in fruit and pericarp development across different post-anthesis stages in multiple litchi cultivars. Our results revealed that all 55 bZIP genes were expressed during fruit development and maturation, with 32 of them exhibiting differential expression across the three cultivars. These findings suggest that bZIP genes are extensively involved in litchi fruit development and maturation, potentially playing significant regulatory roles.

By analyzing the differential expression of bZIP genes during fruit and pericarp development across different post-anthesis stages in various litchi cultivars, we found that *LITCHI017015.m1* exhibited downregulated expression in the pericarp and aril of *Nuomici* and *Huaizha* at 55, 62, and 69 days after flowering (DAF). Similarly, in the cultivar *Feizixiao*, this gene also exhibited downregulated expression during the 75–85 DAF fruit development stage but exhibited upregulated expression during the 35–60 DAF stage. Meanwhile, *LITCHI004463.m1* exhibited upregulated expression during the 35–69 DAF fruit development stage but exhibited downregulated expression in the pericarp and aril of *Nuomici* and *Huaizha* at 55, 62, and 69 DAF. These findings suggest that both genes may play roles in the early stages of fruit development. The qRT-PCR results indicate that the expression levels of genes *LITCHI004463.m1* and *LITCHI017015.m1* are significantly upregulated during the early stages of fruit development, further confirming the above hypothesis.

Similar phenomena have been reported in other plant species. For example, the bZIP gene *npr1* in tomatoes exhibits upregulated expression during the development of locular tissue and mesocarp at 20 DAF [57], while the *MabZIP* gene in bananas plays an essential role in the early stages of fruit development [58]. In carrots, *CAREB1* is an important bZIP transcription factor that regulates gene expression during late embryonic development, dormancy, and maturation [59]. Likewise, our study found that *LITCHI018843.m1* exhibited upregulated expression during the late fruit development stage (40–85 DAF). Moreover, this gene exhibited upregulated expression in the pericarp and aril of *Nuomici* and *Huaizha* at 40, 55, 62, and 69 DAF. In addition, qRT-PCR validation results further confirmed that the expression level of *LITCHI018843.m1* is significantly upregulated during the later stages of fruit development, suggesting that *LITCHI018843.m1* may play a regulatory role in the later stages of litchi fruit development.

## 4. Materials and Methods

### 4.1. Identification of the bZIP Gene Family

In this study, the genome sequence data for litchi were obtained from the Litchi Genome Database [53]. We retrieved the gene IDs of the bZIP transcription factor family and downloaded the corresponding gene sequences, protein sequences, 2000 bp upstream promoter sequences, and genome annotation files. Additionally, the bZIP gene sequences for *Arabidopsis thaliana* were obtained from the Arabidopsis Information Resource (TAIR) (https://www.arabidopsis.org/, accessed on 18 April 2025).

### 4.2. Protein Physicochemical Property Analysis and Subcellular Localization

Using the ProtParam tool from the ExPASy online analysis platform (https://web.expasy.org/protparam/, accessed on 19 April 2025) [60], we conducted a detailed analysis of the physicochemical properties of litchi bZIP transcription factors, including amino acid count, molecular weight, isoelectric point (pI), hydrophilicity/hydrophobicity, aliphatic index, and instability index. Additionally, the subcellular localization was predicted using Plant-mPLoc from the Cell-PLoc 2.0 online tool (http://www.csbio.sjtu.edu.cn/bioinf/plant-multi/, accessed on 16 April 2025) [61], based on their amino acid sequences.

### 4.3. Protein Structure Prediction and Analysis and Protein–Protein Interaction Network Analysis

We employed the online service SOPMA (https://npsa-prabi.ibcp.fr/cgi-bin/npsa_automat.pl?page=npsa%20_sopma.html, accessed on 20 April 2025) to predict the secondary structure of proteins in the litchi bZIP gene family. The corresponding stacked graphs were plotted using Excel software for visualization. Additionally, the tertiary structure modeling of litchi bZIP transcription factors was performed using the SWISS-MODEL tool available on the ExPASy [60] online platform (https://beta.swissmodel.expasy.org/, accessed on 18 April 2025). The modeling results were further assembled and presented using PowerPoint software.

To investigate potential protein–protein interactions among bZIP gene family members, we conducted protein interaction network modeling using the STRING online database (https://cn.string-db.org/, accessed on 23 April 2025), referencing data from the Arabidopsis protein database. This analysis provided insights into the functional interactions among proteins within the litchi bZIP gene family.

### 4.4. Phylogenetic Analysis of the Gene Family

Multiple sequence alignments of the amino acid sequences and conserved domains of 78 *Arabidopsis thaliana* and 55 *Litchi* bZIP genes were performed using MEGA 7.0 software [62]. Phylogenetic clustering was conducted using the Neighbor-Joining (NJ) method, with 1000 bootstrap replicates to assess the reliability of the results. The evolutionary distances were calculated based on the Poisson correction model. Finally, the exported phylogenetic tree in nwk format was refined and visually enhanced using the ITOL online tool (https://itol.embl.de/, accessed on 25 April 2025).

### 4.5. Conserved Domain, Gene Structure, and Motif Analysis of the Gene Family

We utilized the Simple MEME Wrapper function in Tbtools v2.225 [63] software to predict and analyze the conserved domains of bZIP protein sequences, setting the number of motifs to 10 while keeping other parameters at their default values. Subsequently, gene structure analysis was performed using the Gene Structure View (Advanced) function in TBtools [63], incorporating the GFF gene structure file of *Litchi*.

### 4.6. Chromosomal Localization and Intra- and Interspecific Synteny Analysis of the Gene Family

The chromosomal localization of the Litchi bZIP gene family was determined using the Gene Location Visualize from GTF/GFF function in TBtools [63], by integrating gene IDs with the litchi GFF annotation file. Intraspecific synteny analysis was performed using BLASTp. The results were visualized using the Circle Gene View function in TBtools and further refined using Adobe Illustrator. For interspecific synteny analysis, we utilized JCVI v1.4.19 software [64] to compare bZIP genes among six species in the Sapindaceae family. Blue lines represent syntenic relationships among bZIP genes.

### 4.7. Promoter Analysis of the Gene Family

The 2000 bp upstream promoter sequences of *Litchi* bZIP genes were extracted from the *Litchi* genome dataset [53] and submitted to PlantCARE (https://bioinformatics.psb.ugent.be/webtools/plantcare/html/, accessed on 25 April 2025) for cis-regulatory element analysis. After filtering and integrating the analysis results, the Simple BioSequence Viewer function in TBtools [63] was used for visualization.

### 4.8. Tissue Expression Patterns of Litchi bZIP Genes and Differential Expression Analysis During Fruit Development

To analyze the expression patterns of bZIP genes in *Litchi chinensis*, we collected transcriptome data from different tissues (Project ID: PRJNA747875) [53]. Additionally, we integrated multiple transcriptome datasets, including transcriptome data from the pericarp and aril during fruit development and cracking (Project ID: PRJNA681070), with a total data volume of 483 Gb [65]; transcriptome data of litchi fruits in different developmental stages after flowering (Project ID: PRJNA883943), with a total data volume of 72 Gb [66]; and transcriptome data from the pulp of the “Feizixiao” cultivar at 35, 63, and 69 days after flowering (Project ID: PRJNA906072), with a total data volume of 61 Gb [67]. The raw sequencing data were subjected to quality control using Trimmomatic (v0.32) [68] and subsequently aligned to the reference genome with HISAT2 (v2.1.0) under default parameters [69]. Gene expression levels were quantified using Cufflinks, generating FPKM (Fragments Per Kilobase of transcript per Million mapped reads) values to assess gene expression and identify differentially expressed genes (DEGs) [70]. To visually represent the expression patterns, TBtools Heatmap Illustrator (v1.123) [63] was used to generate FPKM-based heatmaps, while EVenn was employed for the visualization of differentially expressed genes [71].

### 4.9. Fluorescence Quantitative Verification

Total RNA samples from litchi fruit, stored at −80 °C in our laboratory, were extracted at 20, 40, and 60 days after flowering, followed by cDNA synthesis and gDNA removal. The resulting cDNA was used as the template for qRT-PCR experiments with 3 biological repetitions. The reaction system consisted of 10 μL of ArtiCanATM SYBR qPCR Mix, 0.4 μL of forward and reverse primers each, 1 μL of the template, 0.4 μL of 50 × ROX Reference Dye I/II, and 7.8 μL of ddH2O. The reaction program was set as follows: 95 °C for 1 min for pre-denaturation, 95 °C for 10 s for denaturation, 60 °C for 20 s for annealing, and 72 °C for 15 s for extension, with a total of 40 cycles. After the experiment, the running data were collected and analyzed using the 2^−△△Ct^ method, followed by compiling and generating the data analysis report. The sequences of the fluorescence quantitative primers are listed in Supplementary Table S5.

## 5. Conclusions

This study systematically identified the molecular characteristics and functional properties of the bZIP gene family in *Litchi chinensis* through bioinformatics analysis. A total of 55 bZIP gene family members were identified in the *Litchi* genome and classified into 13 subfamilies. These genes were successfully mapped onto 15 chromosomes, demonstrating high evolutionary conservation and intraspecific collinearity. Furthermore, expression analysis across different cultivars, tissues, and fruit developmental stages, along with qRT-PCR validation, indicated that *LITCHI017015.m1* and *LITCHI004463.m1* may be involved in the early regulation of fruit development, while *LITCHI018843.m1* primarily functions during the later stages of fruit development. The findings of this study provide essential theoretical insights into the evolution, function, and regulatory roles of the bZIP gene family in fruit development. Moreover, they lay a foundation for genetic improvement and environmental adaptability research in *Litchi*, highlighting its potential applications.

## Data Availability

No new data were created or analyzed in this study.

## Supplementary Materials

The following supporting information can be downloaded at: https://www.mdpi.com/article/10.3390/plants14101453/s1.
