# Supplementary figures and images for: Comprehensive Genome-Wide Investigation and Transcriptional Regulation of the bZIP Gene Family in Litchi Fruit Development
Source: Plants (Basel). 2025 May 13;14(10):1453. doi: 10.3390/plants14101453 (PMC12115174; doi:10.3390/plants14101453)

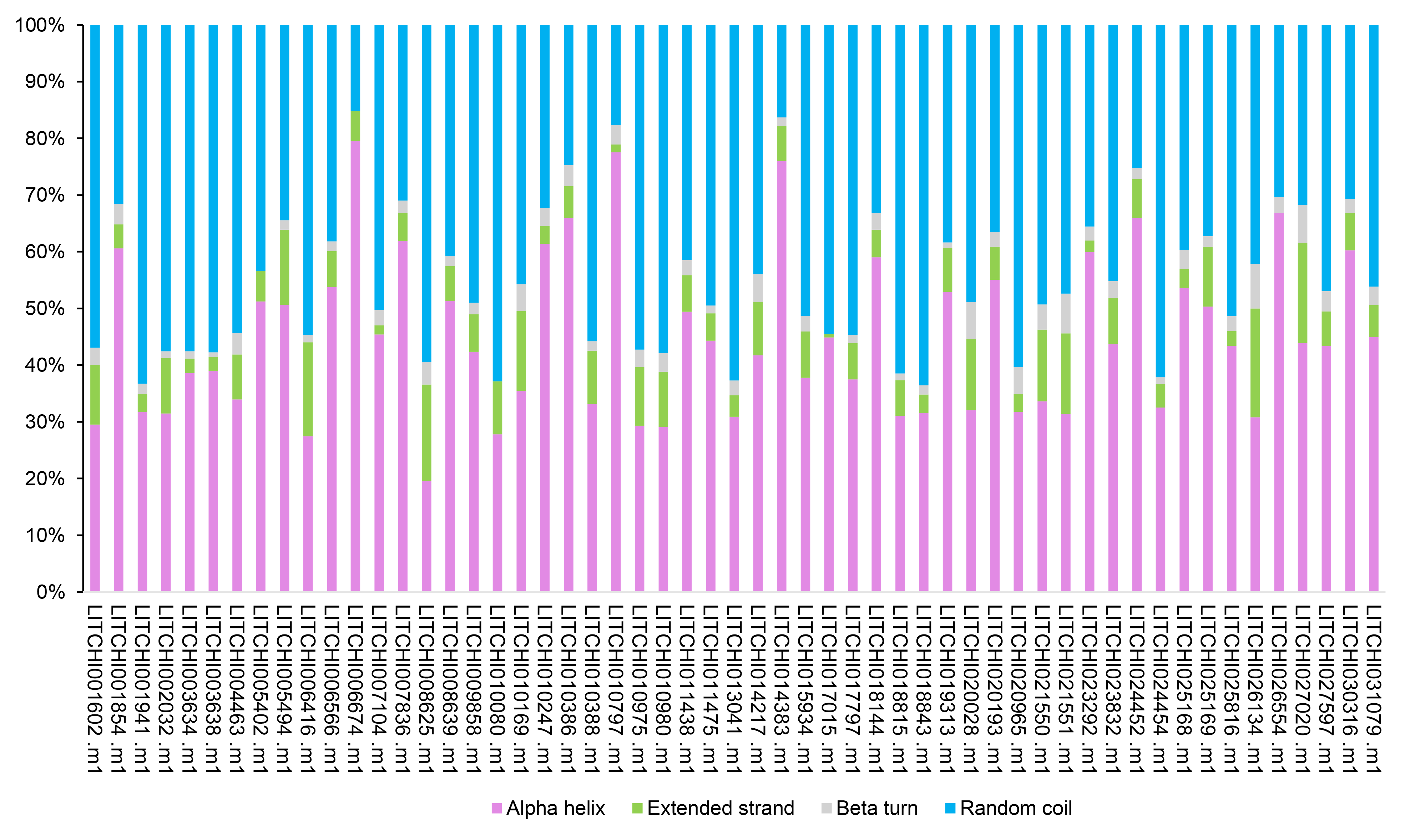

Supplement: Supplementary file 1 [file plants-14-01453-s001.zip › Figure S1.tif]

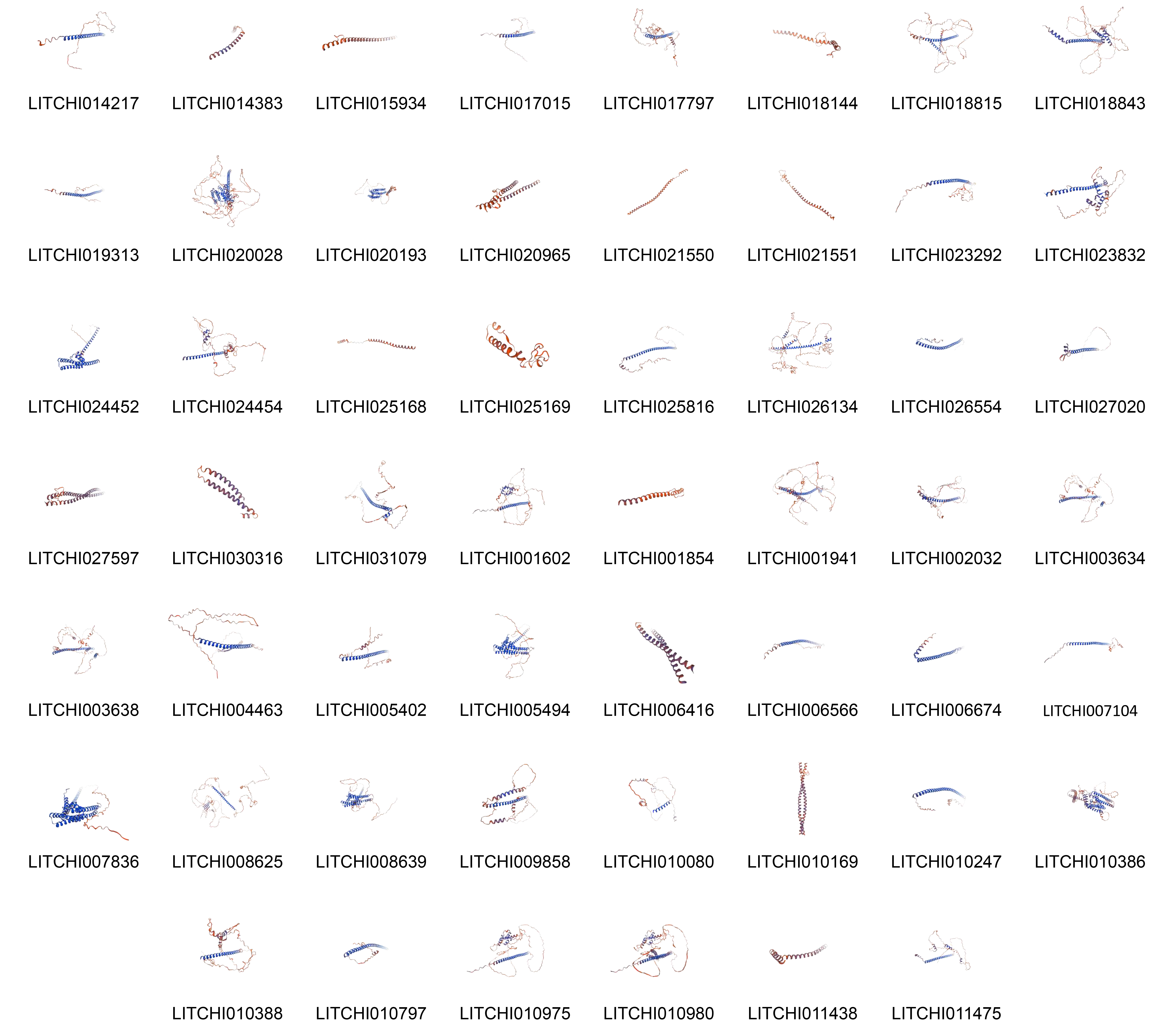

Supplement: Supplementary file 1 [file plants-14-01453-s001.zip › Figure S2.tif]

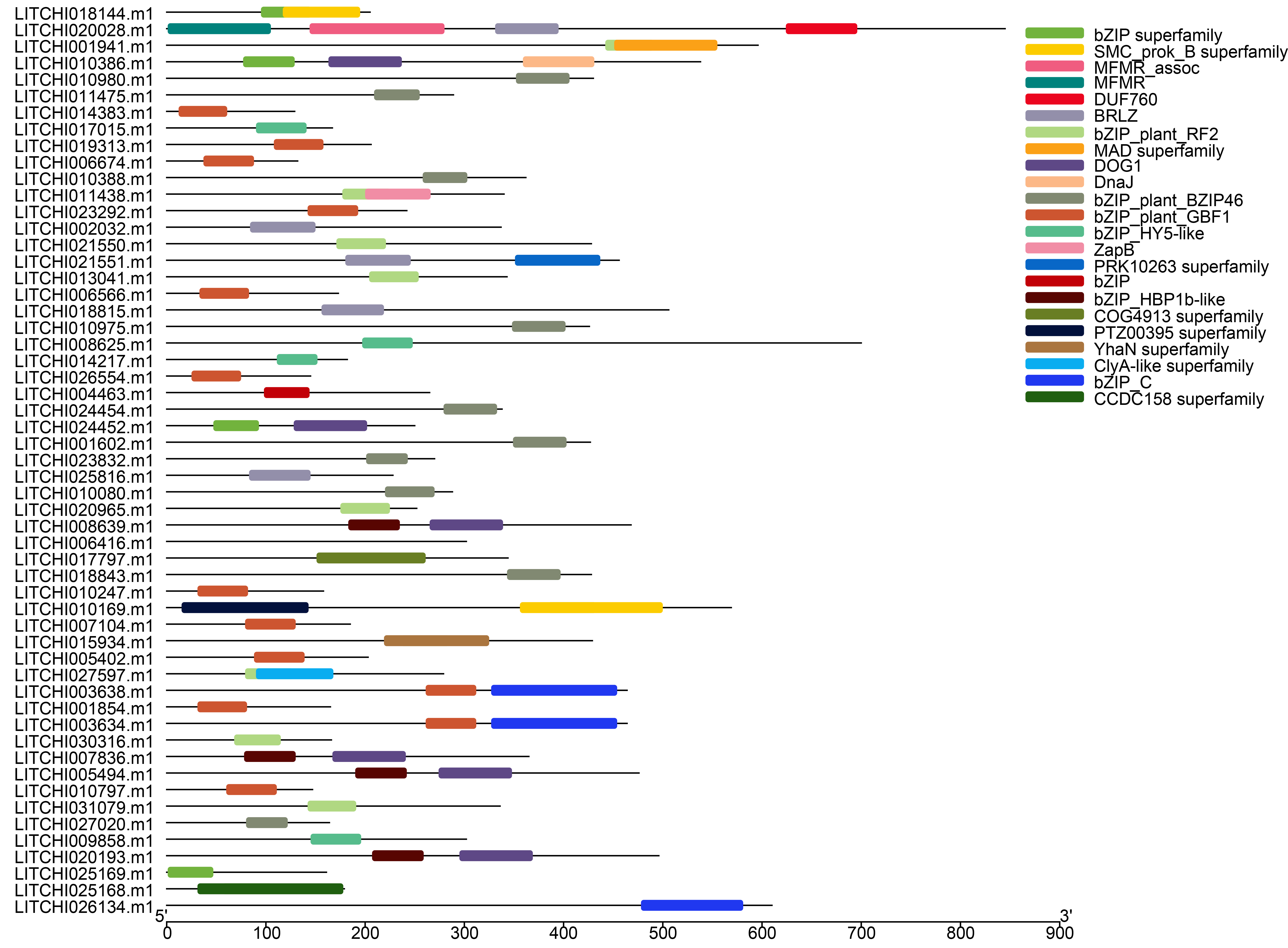

Supplement: Supplementary file 1 [file plants-14-01453-s001.zip › Figure S3.tif]

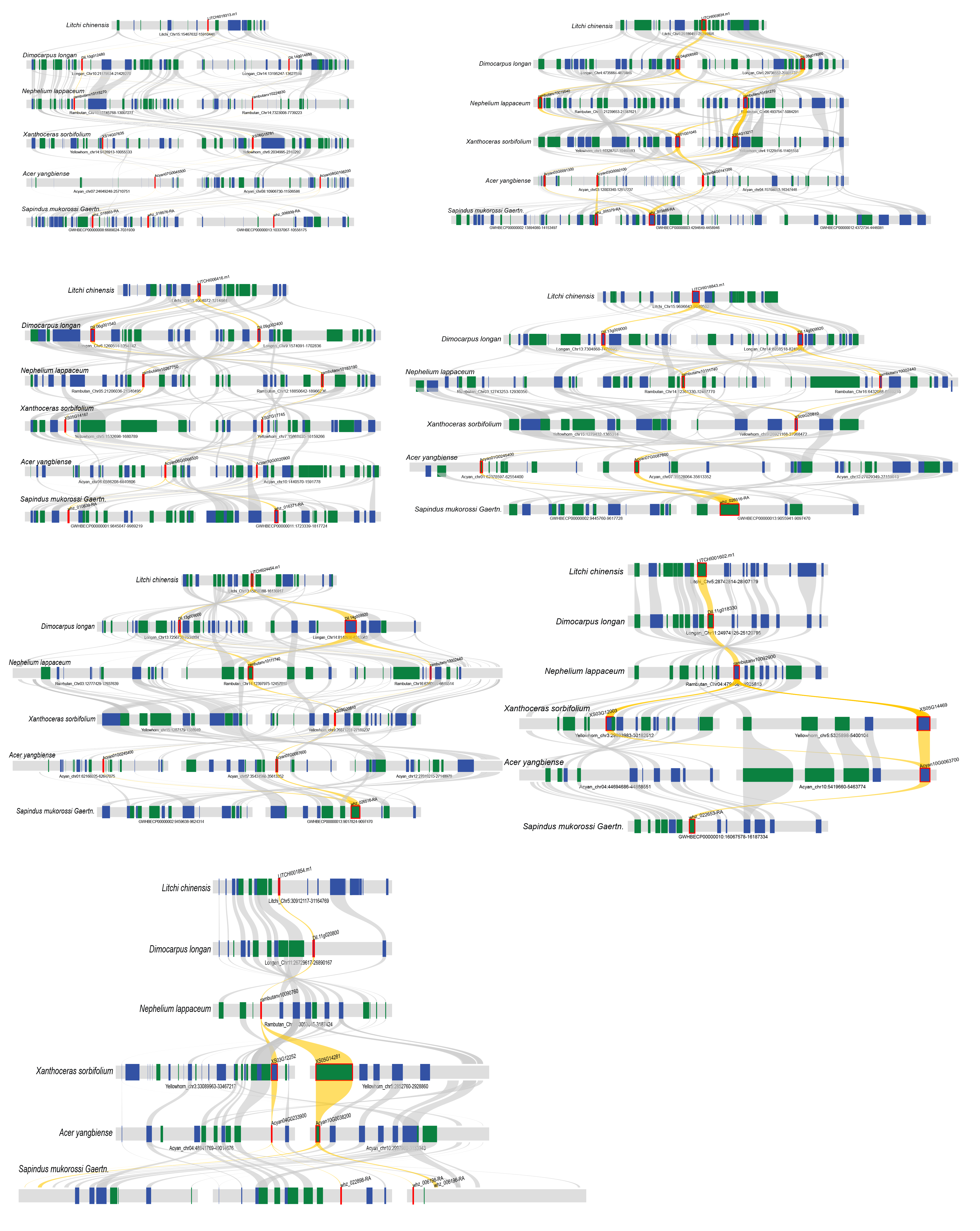

Supplement: Supplementary file 1 [file plants-14-01453-s001.zip › Figure S4.tif]

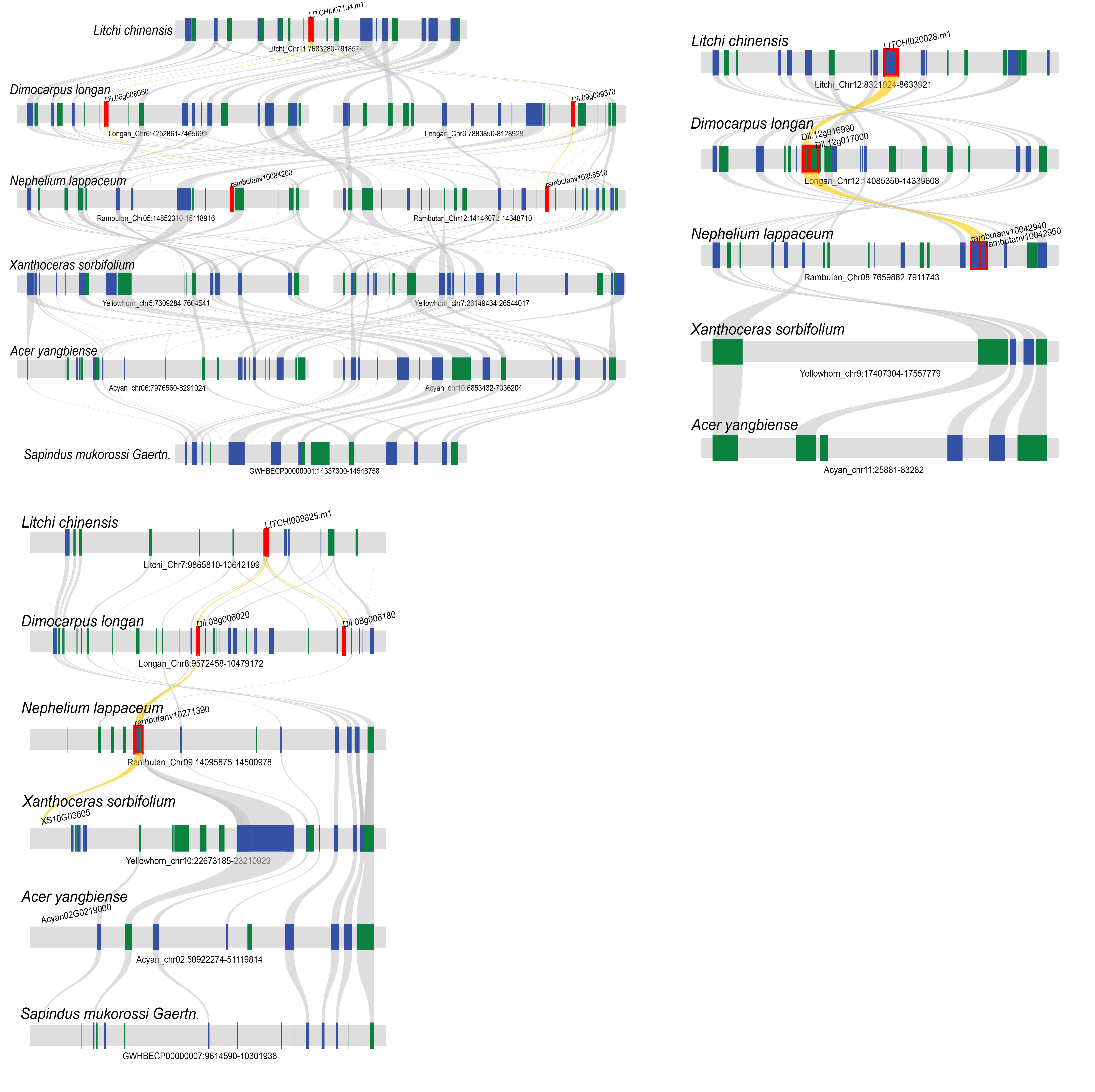

Supplement: Supplementary file 1 [file plants-14-01453-s001.zip › Figure S5.tif]

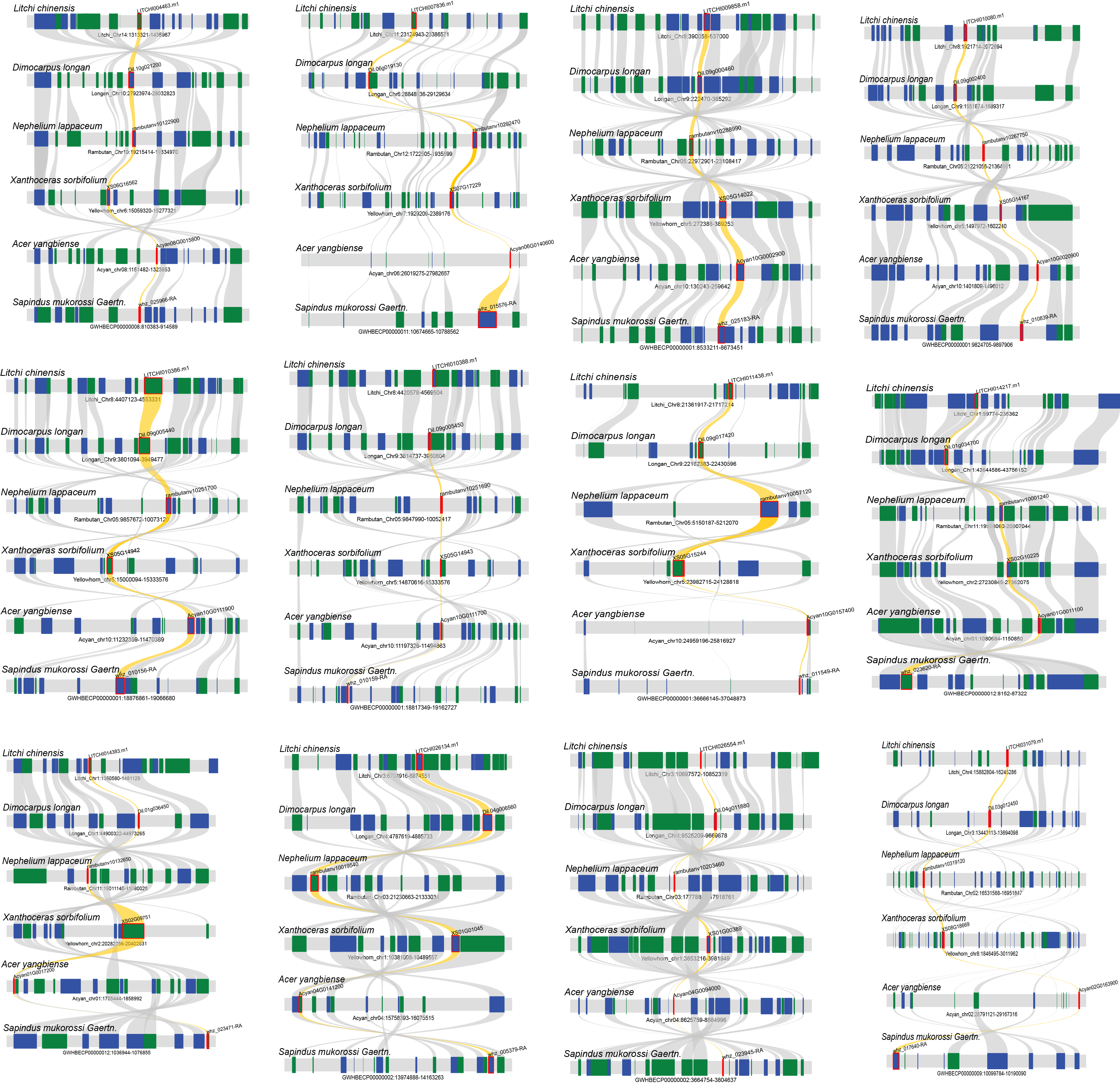

Supplement: Supplementary file 1 [file plants-14-01453-s001.zip › Figure S6.tif]

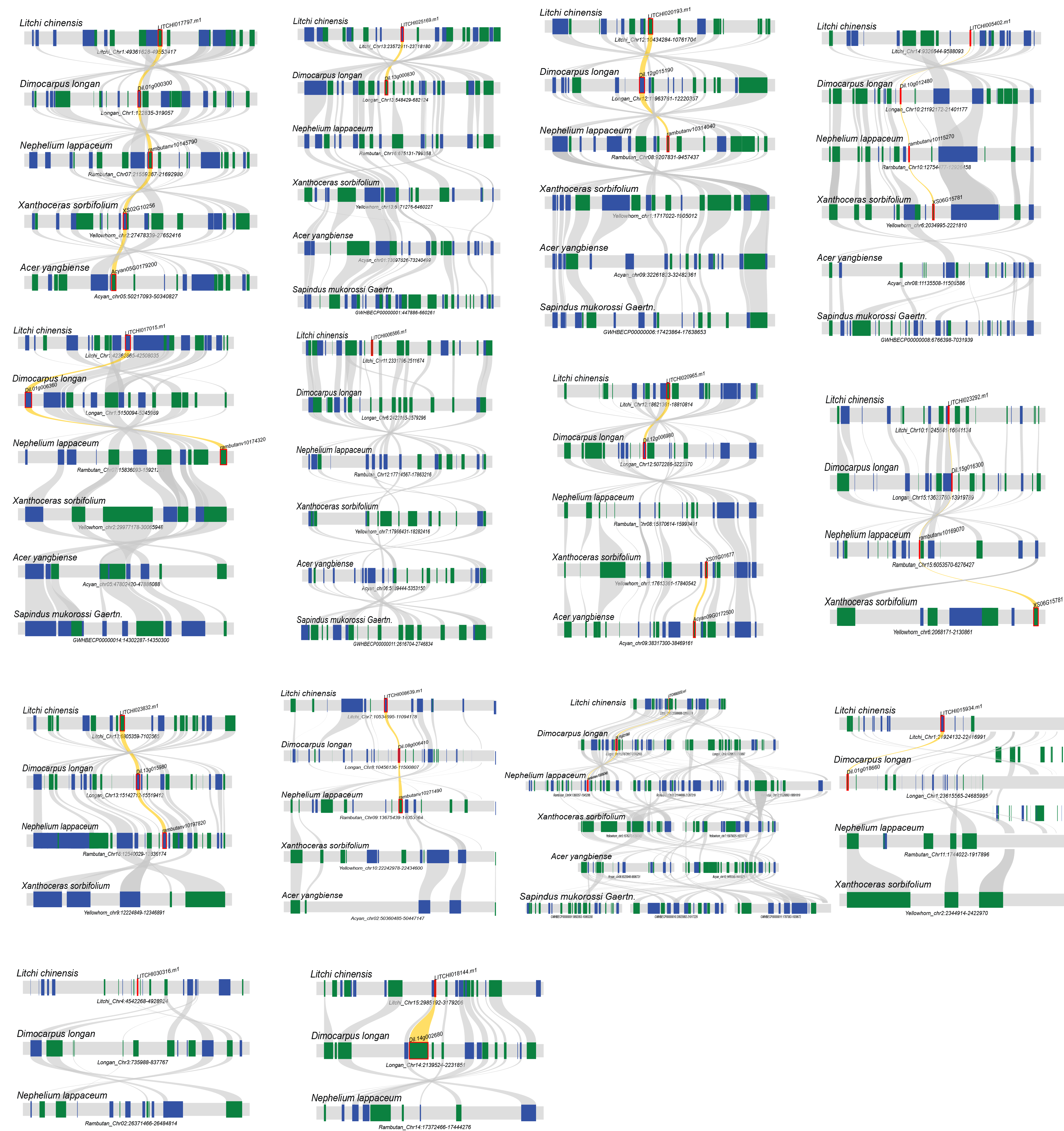

Supplement: Supplementary file 1 [file plants-14-01453-s001.zip › Figure S7.tif]

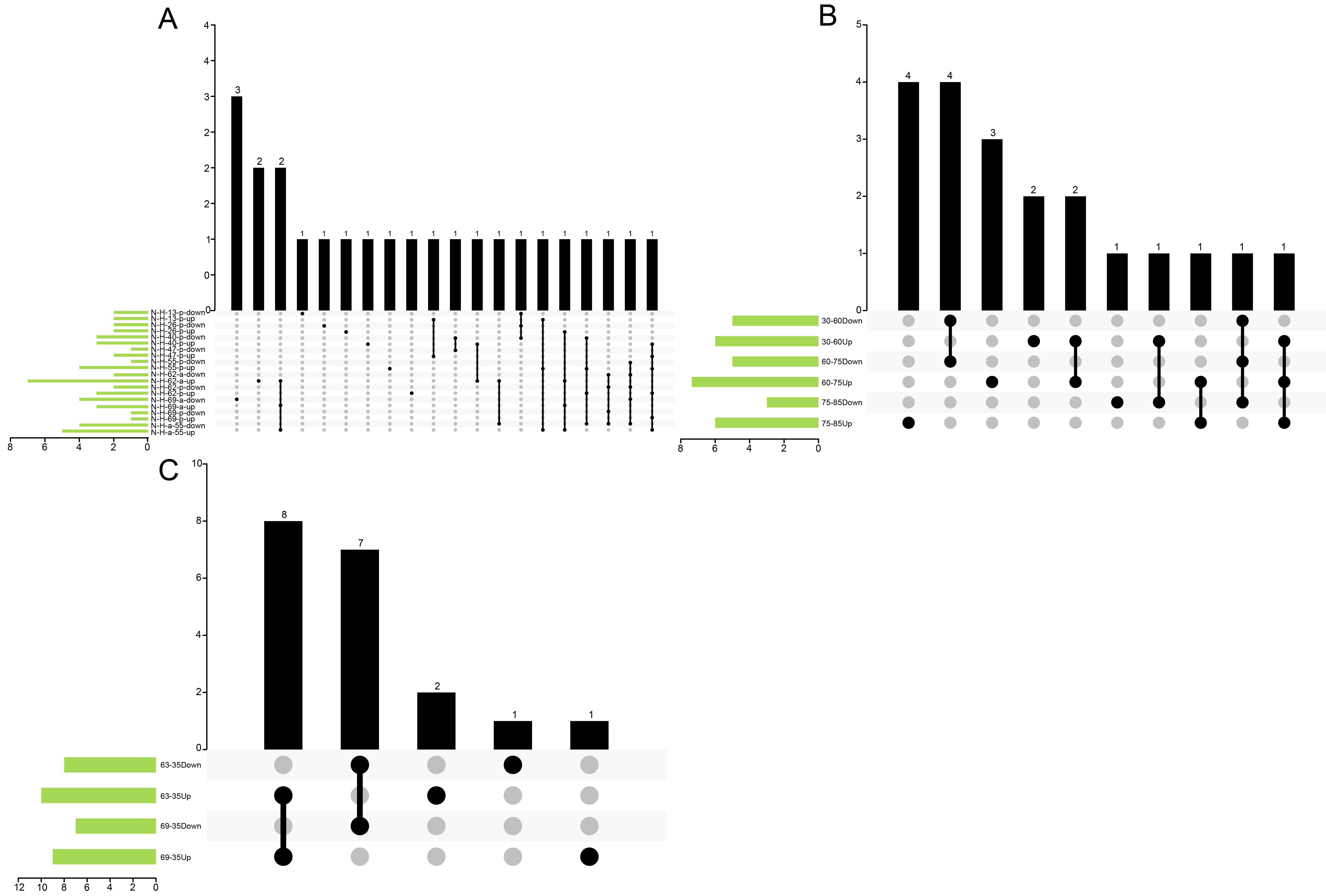

Supplement: Supplementary file 1 [file plants-14-01453-s001.zip › Figure S8.tif]
